# Hsp70 Cochaperones HspBP1 and BAG-1M Differentially Regulate Steroid Hormone Receptor Function

**DOI:** 10.1371/journal.pone.0085415

**Published:** 2014-01-14

**Authors:** Regina T. Knapp, Michael J. H. Wong, Lorenz K. Kollmannsberger, Nils C. Gassen, Anja Kretzschmar, Jürgen Zschocke, Kathrin Hafner, Jason C. Young, Theo Rein

**Affiliations:** 1 Max Planck Institute of Psychiatry, Chaperone Research Group, Munich, Germany; 2 McGill University, Department of Biochemistry, Groupe de Recherche Axé sur la Structure des Protéines, Montreal, Canada; University of Pittsburgh, United States of America

## Abstract

Hsp70 binding protein 1 (HspBP1) and Bcl2-associated athanogene 1 (BAG-1), the functional orthologous nucleotide exchange factors of the *heat shock protein 70 kilodalton* (Hsc70/Hsp70) chaperones, catalyze the release of ADP from Hsp70 while inducing different conformational changes of the ATPase domain of Hsp70. An appropriate exchange rate of ADP/ATP is crucial for chaperone-dependent protein folding processes. Among Hsp70 client proteins are steroid receptors such as the glucocorticoid receptor (GR), the mineralocorticoid receptor (MR), and the androgen receptor (AR). BAG-1 diversely affects steroid receptor activity, while to date the influence of HspBP1 on steroid receptor function is mostly unknown. Here, we compared the influence of HspBP1 and BAG-1M on Hsp70-mediated steroid receptor folding complexes and steroid receptor activity. Coimmunoprecipitation studies indicated preferential binding of Hsp40 and the steroid receptors to BAG-1M as compared to HspBP1. Furthermore, Hsp70 binding to the ligand-binding domain of GR was reduced in the presence of HspBP1 but not in the presence of BAG-1M as shown by pull-down assays. Reporter gene experiments revealed an inhibitory effect on GR, MR, and AR at a wide range of HspBP1 protein levels and at hormone concentrations at or approaching saturation. BAG-1M exhibited a transition from stimulatory effects at low BAG-1M levels to inhibitory effects at higher BAG-1M levels. Overall, BAG-1M and HspBP1 had differential impacts on the dynamic composition of steroid receptor folding complexes and on receptor function with important implications for steroid receptor physiology.

## Introduction

Efficient protein folding is a major prerequisite for correct biological function and cellular protein homeostasis. About 20% of the eukaryotic proteins are clients of the Hsp70-Hsp90 chaperone machinery (constitutively expressed Hsc70 and inducible Hsp70 are almost identical and collectively referred to here as Hsp70 unless specifically stated) [1]. Among the clients of Hsp70-Hsp90, nuclear receptors such as the glucocorticoid receptor (GR), the mineralocorticoid receptor (MR), and the androgen receptor (AR) take a prominent role. These receptors rely on folding assistance by (co)chaperones to acquire hormone binding capacity and transcriptional activity [2], [3]. Compromised receptor folding might lead to impaired cellular and physiological function, and thus to pathology. For instance, there is ample evidence that misfolding and malfunctioning of GR is involved in the pathogenesis of major depressive disorder [4], [5].

In addition to the core chaperones Hsp70 and Hsp90, cochaperones like Hsp40, Hsp70-interacting protein (Hip), Hsp-organizing protein (Hop), diverse tetratricopeptide repeat (TPR) domain proteins, and p23 also play important roles during steroid receptor folding [6], [7]. Hsp70, Hsp90, and their cochaperones operate in a step-wise and combinatorial fashion [8], [9]. Hsp40 is thought to initially bind to the nascent receptor for delivery to Hsp70 [10], [11]. Moreover, Hsp40 promotes hydrolysis of ATP by Hsp70 to facilitate tight binding of the chaperone to the substrate [10], [12]. The Hsp70-receptor-complex is stabilized by Hsp70 interacting protein (Hip), preventing ADP dissociation from Hsp70 [13]. Hop is thought to bridge Hsp70 and Hsp90, thereby allowing transfer of the client protein to Hsp90. As a cofactor of Hsp90, p23 is found at late stages of steroid receptor folding after dissociation of Hsp70 and Hop. It eventually stabilizes the ATP-bound Hsp90-receptor complex and acts as a substrate releasing factor of Hsp90 [14].

Hydrolysis of ATP and exchange of ADP for ATP are key processes for substrate binding and release by Hsp70 to keep the folding cycle operative. Nucleotide exchange can be mediated by Bcl-2-associated athanogene 1 (BAG-1) and Hsp70 binding protein 1 (HspBP1), both of which bind to the ATPase domain of Hsp70 as nucleotide exchange factors (NEFs). They have been reported to both positively and negatively modulate Hsp70 chaperone activity [7], [15], [16].

Mammalian cells express four translational BAG-1 isoforms: a large (BAG-1L), a medium (BAG-1M), a short (BAG-1S), and a very short isoform designated p29 [17]. These isoforms share a C-terminal BAG domain mediating the interaction with the ATPase domain of Hsp70 and an *ubiquitin-like* domain (ULD) located in the center of the protein [18]. The latter connects BAG-1 to the proteasome and is thought to be involved in CHIP - (E3 ligase) dependent proteasomal degradation of GR [19], [20].

While the isoforms of BAG-1 have a similar effect on the ATPase activity of Hsp70, they differ in their impact on Hsp70-dependent protein refolding [21]. Because effects of BAG-1 isoforms on client protein folding are not directly predictable from their NEF function, clients must be analyzed individually. In fact, isoform-specific effects of BAG-1 have also been observed for the activity of GR, AR, and MR [22]–[25]. For example, the short isoform BAG-1S exhibits no effect on GR, while BAG-1M and the low abundance isoform BAG-1L inhibits GR [24], [25]. The nucleotide exchange of Hsp70 catalyzed by HspBP1 is based on a different molecular mechanism compared to BAG-1M [16]. A comparison of BAG-1 and HspBP1 revealed that they have different structures, bind to different sites on Hsp70, and induce different conformational changes of the ATPase domain of Hsp70 in the nucleotide-free state [16], [26]. This might lead to different effects on some Hsp70 clients.

BAG-1 and HspBP1 have been recently described to cooperate differently with the Hsp40 homolog Hdj3 (DJA2/DNAJA2) in Hsp70-dependent refolding [7]. However, the impact of HspBP1 on steroid receptor function has not been elucidated yet.

Here, we compared the influence of HspBP1 and BAG-1M on the composition of chaperone-steroid receptor heterocomplexes, substrate binding ability of Hsp70, and transcriptional activity of GR, MR, and AR.

## Materials and Methods

### Antibodies

Antibodies purchased from Santa Cruz Biotechnology (California, USA) were anti-actin (I-19), anti-Hsc70 (B-6), anti-Hsp90 (H-114), anti-BAG-1 (C-16), and anti-goat; antibodies from Stressgen (British Columbia, Canada) were anti-Hip (SPA-766), anti-Hop (SRA-1500), and anti-Hsp40 (SPA-450, specific for Hsp40/Hdj1); from Sigma-Aldrich (St. Louis, USA) were anti-Flag-HRP (M2), anti-rabbit, and anti-mouse. Further antibodies were anti-CHIP (Calbiochem, San Diego, USA), anti-HA-HRP (3F10; Roche Diagnostics, Mannheim, Germany), anti-p23 (MA3-414; Affinity Bio Reagents, Golden, USA), anti-His (ab14923-100; Abcam, Massachusetts, USA), anti-HspBP1 (MAB-10201, Biozol, Eching, Germany), and anti-rabbit (Cell Signaling, Massachusetts, USA).

### Plasmids and Proteins

The plasmids expressing the N-terminally HA-tagged receptors GR, MR, and AR (pRK7 backbone) were kindly provided by A. Hoffmann (laboratory of D. Spengler, MPI of Psychiatry, Munich). The Gaussia-KDEL control plasmid [6], the MMTV-Luc reporter plasmid [27], [28], and the BAG-1 expressing plasmids have been described previously [25]. The N-terminally Flag-tagged HspBP1 or HspBP1_mut (A137R, K249A [16]) expressing plasmids were constructed by PCR amplification from pet28a-HspBP1 [15] and insertion of the amplified cDNA into the vectorpRK5MCS. For protein purification, cDNAs of human BAG-1M_mut and HspBP1_mut were ligated into pProExHta. Cloning and sequence details are available on request. Purification of HspBP1, BAG-1M, Hsc70, and GR-LBD proteins has been described previously [16].

### Electroporation and Coimmunoprecipitation

Protocols for cultivation of HEK293 cells, electroporation, and immunoprecipitation of Flag-tagged BAG-1 and HspBP1 were as described [6], [23], with minor modifications. Plasmids encoding HA-tagged steroid receptor (2 µg) and either Flag-BAG-1M, Flag-BAG-1M_mut, Flag-HspBP1, or Flag-HspBP1_mut (each 8 µg) were combined in one electroporation cuvette. As control for unspecific binding to the Flag-agarose beads, “empty” pRK5MCS vector (8 µg) and HA- receptor plasmids (2 µg) were transfected. Cells were harvested 60–68 h post electroporation and processed for immunoprecipitation. 25 µl of anti-Flag M2 agarose were used for 1.5 mg cellular extract in a final volume of 1 ml. As in previous studies [6], [23], ATP was not added to the binding reaction to more closely represent the starting cellular conditions. Elution was performed with 50 µl of Flag-peptide (100 ng/µl; Sigma Aldrich, St. Louis, USA). Eluates were adjusted to a final volume of 65 µl with Laemmli buffer, and 15 µl or 1.6 µl were loaded on SDS gels for immunoblotting or colloidal Coomassie staining, respectively.

### Western Blot and Relative Protein Quantification

Immunodetection of proteins by Western blotting was carried out as described [23], [29], [30]. The protein signals from chemiluminescence were digitally documented (Image station 440 CF and 1D image analysis software 3.6, both from Kodak, USA).

**Figure 1:**
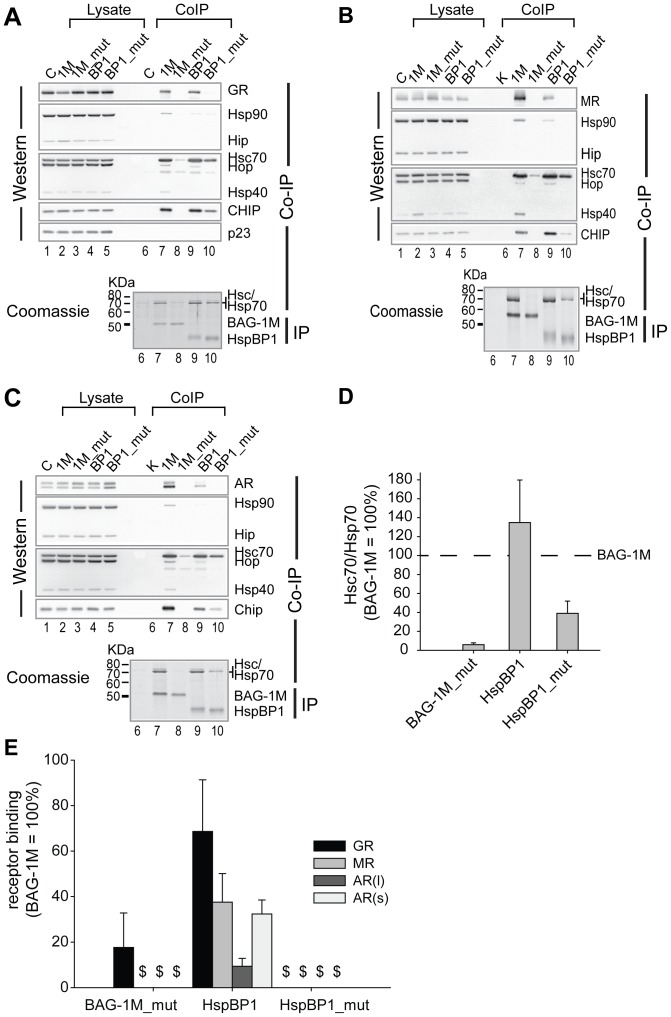
HspBP1 and BAG-1M favor differently composed Hsp70-steroid receptor complexes. HEK293 cells were transiently transfected with the expression plasmids of Flag-tagged wild-type or mutant, Hsp70 binding-impaired BAG-1M or HspBP1, together with 2 µg plasmid expressing either HA-tagged GR, MR, or AR. After immunoprecipitation with anti-Flag agarose, the co-precipitated steroid receptors and endogenous target proteins were analyzed by Western blot. (A–C) Representative Western blots of the input control (lysate) and the co-precipitated proteins are indicated for the experiments with GR (A), MR (B), and AR (C). The precipitated nucleotide exchange factors were visualized by colloidal Coomassie (respective lower panels). (D, E) Comparison of Hsc70/Hsp70 (D) or steroid receptor (E) binding to wild-type and mutant HspBP1 and BAG-1M. The quantity of binding to BAG-1M was used as reference and set to 100%. Shown are the mean values (+SEM, N = 3). (E) In the case of AR, a long (l) and a short (s) form were quantified separately. $ denotes not detectable binding.

For comparison of the relative amount of co-precipitated proteins, the signal of bound receptor was normalized to both the signal of its corresponding input control and the signal of the immunoprecipitated Flag-tagged BAG-1M or HspBP1 or the respective mutants (Fig. 1E, derived from experiments presented in Fig. 1A–C). Signals of the endogenous co-precipitated Hsc/Hsp70 were normalized to the immunprecipitated Flag-proteins (Fig. 1D, derived from experiments presented in Fig. 1A–C). Densitometric scans of Coomassie stained, immunprecipitated Flag-proteins and co-precipitated Hsc/Hsp70 were obtained with a GS-800 calibrated densitometer and PDQuest software (BioRad, Munich, Germany), followed by processing with the 1D image analysis software as above.

**Figure 2:**
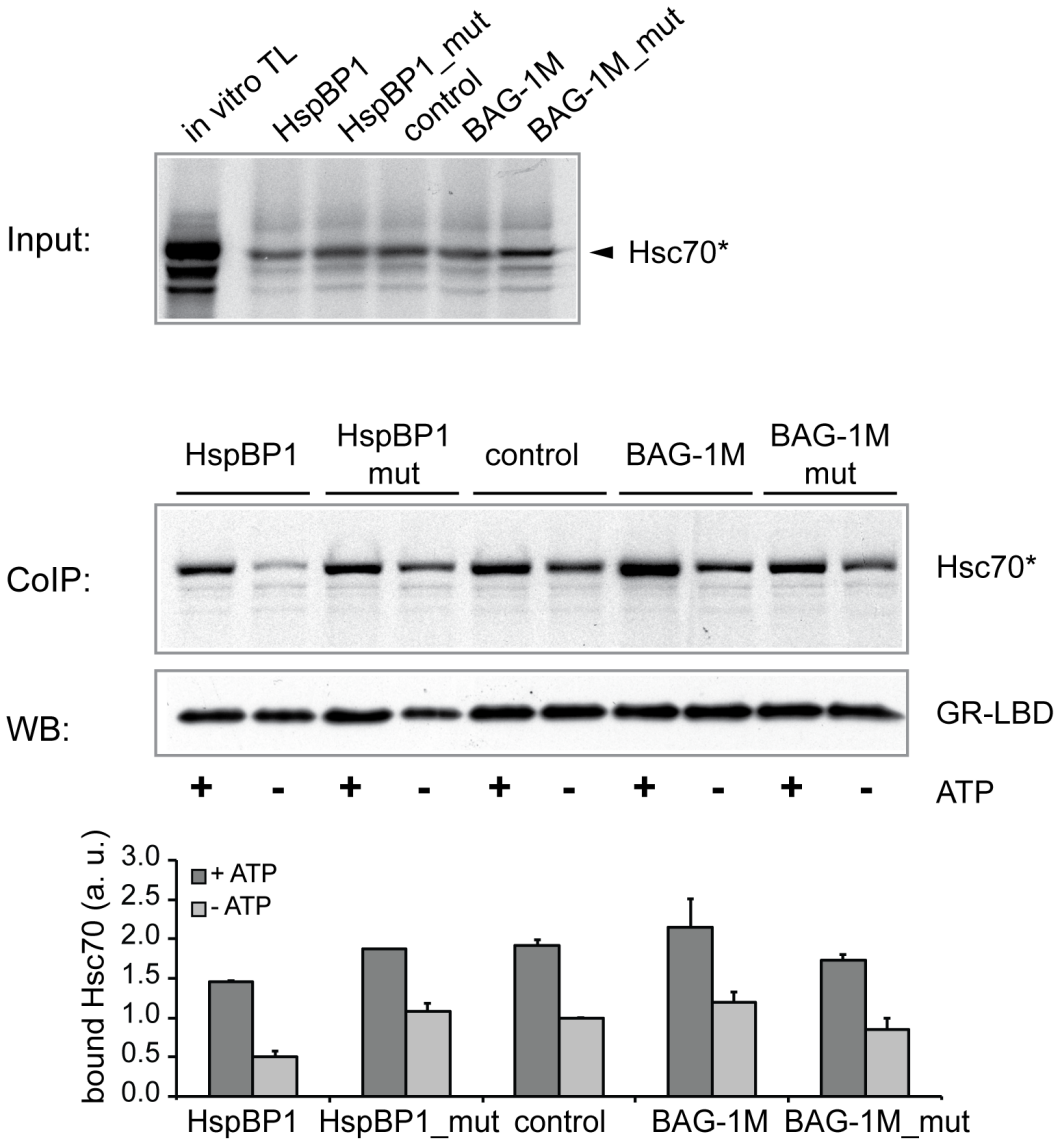
HspBP1 reduces Hsc70 binding to GR-LBD. Using chaperone-binding (pull-down) assays, the effect of HspBP1 and BAG-1M on Hsc70 binding to immobilized GR-LBD was analyzed with purified NEFs and *in vitro* translated and labeled Hsc70 in the presence or absence of ATP. Representative images of labeled Hsc70 (top, input; bottom, co-precipitation) and immuno-detected GR-LBD (after elution) are shown. The bar graph indicates the mean values of bound Hsc70 (+SEM; N = 4). The mean value of bound Hsc70 in the control sample absence of ATP) was set to 1.

In Figure 2, the relative amounts of bound Hsp70, the quantity of anti-c-myc precipitated GR-LBD, and the amount of bound Hsp70, were analyzed with Image J. The density of bound Hsp70 (ratio of bound Hsp70 to input Hsp70) was set as a ratio with precipitated GR-LBD. Hsp70 binding was analyzed in four independent experiments.

### Protein Purification

Expression and purification of wild-type or mutant BAG-1M and HspBP1 in BL21pLys bacteria was as described [7] with the following minor modifications. Induction was with 0.2 mM isopropyl-β-D-thiogalactopyranoside (IPTG) for 2 h at 37°C. The His-tagged proteins in the supernatant were purified using a 2 ml Ni-NTA-agarose column, and eluted with buffer EB containing 300 mM imidazole, 20 mM KH_2_PO_4_ pH 7.5. In order to adjust the buffer conditions for the chaperone-binding assay, the elution buffer was exchanged by 100 mM KOAc, 20 mM Hepes-KOH pH 7.5, 5 mM MgOAc_2_ using dialysis. In case of HspBP1_mut (Fig. 2), buffers for purification additionally contained 10 mM β-mercaptoethanol.

pMS-LBD-Myc/His induction of protein expression was with 1 mM IPTG at 18°C for 12–16 hours. GR-LBD was solubilized from inclusion bodies with 20 mM Tris-HCl pH 7.5, 500 mM NaCl, 1% NP40, purified on Ni-NTA-agarose, and eluted with buffer EL-LBD (300 mM imidazole, 20 mM Tris-HCl pH 7.5, 1% NP40) as described [14].

### *In vitro* Translation and Chaperone Binding

Coupled *in vitro* transcription and translation of Hsc70 was performed in TNT reticulocyte lysate (Promega, Wisconsin, USA) using pet11a-Hsc70 plasmid DNA and radioactively labeled [^35^S]-Met (Perkin Elmer, Massachusetts, USA) according to manufacturer’s manual. The translational reaction (TL) was incubated for one hour at 30°C.

Immobilized GR-LBD was used as Hsp70 client protein. After denaturation of the GR-LBD in a ten-fold volume of 1% SDS for 10 min at room temperature, refolding was initiated by dilution with 1 ml buffer B (100 mM KOAc, 20 mM HEPES-KOH pH 7.5, 5% glycerol) containing 1% Igepal C-630, 1% sodium deoxycholate, and 0.1% SDS. Anti-c-myc agarose (Sigma Aldrich, St. Louis, USA) in 1xPBS was added to the reaction mixture. The reaction was incubated for one hour at room temperature on a shaker and then washed with buffer B (w/o detergents) for two times.

The chaperone-binding (pull-down) assay was carried out with reticulocyte lysate (Promega, Wisconsin, USA) in the presence of 7 µM purified BAG-1M, BAG-1M_mut, HspBP1, HspBP1_mut, or an equal molarity of BSA as negative control. The lysate was desalted, and the buffer was exchanged against buffer B (100 mM KOAc, 20 mM HEPES-KOH pH 7.5, 5% glycerol) before use. The sodium chloride concentration of the purified proteins and the control (BSA) were adjusted to 70 mM each. Input reactions contained 50% desalted reticulocyte lysate, 4% Hsc70-TL, 5 mM MgOAc_2_, 7 µM of purified NEF or BSA (control without NEF) in buffer B. Each sample was prepared as duplicate. After pre-incubation for 15 min at room temperature, samples were put on ice. After addition of ATP (final concentration 1 mM) to one series of each NEF sample, 200 µl reactions were combined with 40 µl immobilized GR-LBD agarose in buffer B. Samples without ATP were kept on ice, samples with ATP were incubated for 15 min at room temperature. Remaining ATP was removed by addition of 2 units Apyrase (Sigma Aldrich, St. Louis, USA). The agarose was collected by centrifugation and washed two times with 500 µl buffer B. Precipitated LBD and bound Hsc70 were eluted by boiling with SDS-sample buffer (40% glycerol, 160 mM Tris-HCl pH 6.8, 5% β-mercaptoethanol, 5% SDS, 0.025% bromophenole blue). Subsequent to SDS-PAGE, the amount of input and bound Hsc70 was monitored with a phospho-imager. The amount of precipitated GR-LBD was detected by immunoblot analysis using a polyclonal anti-His antibody and ECL substrate. As shown previously [14], the bead material does not pull down Hsp70.

### Reporter Gene Assays

Cultivation of Cos-7 cells in 96-well plates and reporter gene assays were as described [6], [29] with some adaptations. The total amount of plasmid transfected with Exgene was adjusted to 277.5 and 290 ng per well. To obtain comparable levels of receptor expression, 12.5 ng of HA-GR plasmid, and 25 ng of MR and AR encoding plasmids were used in combination with the other plasmids. To study MR activity, Cos-7 cells were grown in 0.5% charcoal-stripped FBS in order to reduce the basal activity of non-stimulated MR [6]. Readings of Firefly luciferase activity were referred to the respective Gaussia activities to compensate for assay variations. In cases when Gaussia activities did not accurately measure transfection efficiency, we made sure that a high enough number of independent Luciferase readings were performed for sufficient statistical strength. The paired t-test was applied for analysis of significance.

To monitor the amount of transiently expressed steroid receptors and cofactors, triplicates (30 µl per well) were pooled for Western blot analysis after determination of the luciferase activities. For MR and AR, it was necessary to concentrate the pooled samples for 30 min at 30°C by vacuum rotation (Concentrator 5301, Eppendorf, Hamburg, Germany). For the detection of AR, 6.5 µl SDS (20%) were added to 50 µl extracts, and sonication was performed, eight times for 30 sec at level 5 (Bioruptor; Diagenode, Liege, Belgium). Samples were boiled for 15 min at 90°C with 15 µl 5x Laemmli sample buffer; 20 µl were used for SDS-PAGE.

### Hsc70 ATPase Assay

The ATPase activity of Hsc70 stimulated by DJA2/DNAJA2, in the presence or absence of purified HspBP1 or HspBP1_mut, was determined as previously described [7]. Concentrations of Hsc70, DJA2 and HspBP1 (wt and mut) were 4 µM and reactions were at 30°C. The paired t-test was used to test for significance (comparing HspBP1 wt and mut).

## Results

### HspBP1 and Hsp40 are not Part of the Same Hsp70-receptor-heterocomplex

We compared the interaction profiles of HspBP1 and BAG-1M with different steroid receptors, Hsp70, and other folding relevant (co)chaperones by coimmunoprecipitation. BAG-1M having a cytosolic/nuclear localization was chosen over nuclear BAG-1L to examine effects in either compartment, and because of its normally high abundance in cells; furthermore, BAG-1M transfection also expressed the shorter isoforms by internal translation initiation, at normal physiologically low levels [17]. Plasmids encoding Flag-tagged wild-type HspBP1 or BAG-1M, or mutant forms defective in Hsp70 binding (HspBP1_mut or BAG-1M_mut) were transiently transfected into HEK-293 cells along with vectors expressing either HA-tagged GR, MR, or AR. The BAG-1M_mut construct R237A was as previously published [25],[31], and HspBP1_mut (A137R, K249A) was derived from the HspBP1-Hsp70 co-crystal structure, and features the same amino acid exchanges as those in the yeast orthologue Fes1p that impaired Hsp70 binding [16].

All three steroid receptors GR, MR, and AR were found to co-precipitate with HspBP1 (lane 9 in Figs. 1 A–C and Fig. 1E, respectively), but not with HspBP1_mut (lane 10 in Figs. 1A–C and Fig. 1E, respectively). A similar Hsp70-dependent receptor interaction profile was observed for wild-type and mutant BAG-1M (lanes 7 and 8 in Figs. 1A–C and Fig. 1E, respectively) though BAG-1M co-precipitated greater amounts of GR, MR, and AR compared to HspBP1 (Figs. 1A–C and 1E), but slightly less Hsc70 and inducible Hsp70 (Figs. 1A–D) than HspBP1. As expected for Hsp70 NEFs, only minor amounts of Hsp90 co-precipitated with BAG-1M, and only traces with HspBP1 (Figs. 1A–C).

A striking difference between HspBP1 and BAG-1M was observed for their interaction profile with Hsp40 (using a monoclonal antibody specific for Hsp40/Hdj1): Hsp40 only co-precipitated with BAG-1M but not with HspBP1 (Figs. 1A–C). CHIP, an E3-ligase, co-precipitated with both HspBP1 and BAG-1M. Small amounts of CHIP were also found to co-precipitate with HspBP1_mut (Fig. 1A–C), likely reflecting residual Hsp70 binding of HspBP1_mut (∼30% of wild-type, Fig. 1D). This residual binding of HspBP1 prompted us to assess its effect on the ATPase activity of Hsc70. HspBP1_mut activated the Hsc70 ATPase significantly less than wild-type HspBP1, although it still exerted a noticeable effect (Table 1) which is in line with its residual binding to Hsc70 (Fig. 1D).

**Table 1:** ATPase activity of purified Hsc70 in the presence or absence of DJA2, HspBP1, or HspBP1_mut, as indicated.

	ATPase (min^−1^)
Hsc70	1.87±1.07
Hsc70+ DJA2	4.50±0.71
Hsc70+ DJA2+ HspBP1	8.02±0.63
Hsc70+ DJA2+ HspBP1_mut	7.26±0.55, *p* = 0.0184 relative to wt

Errors represent standard deviations from the mean.

### HspBP1 Reduces Client Binding of Hsp70

The observed reduced binding of steroid receptors to HspBP1 compared with BAG-1M (Fig. 1E) is possibly due to differences in the impact of the two NEFs on the Hsp70-steroid receptor interaction. We therefore investigated the effects of purified NEFs on Hsp70 binding to the ligand binding domain (LBD) of GR as client protein, with and without added ATP. We performed pull-down assays using *in vitro* translated and labeled Hsc70, purified wild-type and mutant HspBP1 and BAG-1M, respectively, and rabbit reticulocyte lysate lacking an energy regeneration system [7]. *In vitro* translated Hsc70 was incubated with wild-type NEFs or the respective mutants before adding the client protein, i.e., GR-LBD immobilized on agarose beads. We observed an overall greater binding of Hsc70 to the GR-LBD when ATP was added (Fig. 2). In the presence of HspBP1, less Hsc70 was bound to the GR-LBD than in control reactions without NEF. This difference was more pronounced under ATP-free conditions where NEF binding is optimal [16]. BAG-1M slightly increased the interaction between Hsc70 and GR-LBD, while the Hsp70 binding-defective mutants of HspBP1 and BAG-1M exerted no effect (Fig. 2).

### HspBP1 and BAG-1M Divergently Affect Steroid Receptor Activity

To test whether the differences of BAG-1M and HspBP1 on steroid receptor-chaperone heterocomplex interactions and composition translates into altered steroid receptor activity, we performed reporter gene assays with GR, MR, and AR in Cos-7 cells. We varied both the amount of co-transfected BAG-1M or HspBP1 and the concentration of hormone.

**Figure 3:**
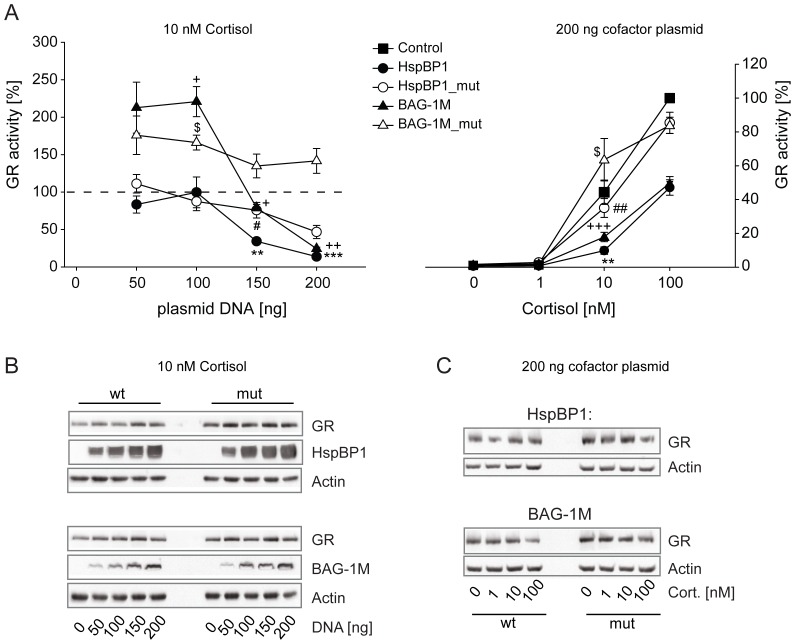
Effects of HspBP1 and BAG-1M on GR transactivation. The impact of HspBP1 and BAG-1M on GR function was assessed by reporter gene experiments in Cos-7 cells. Control and reporter plasmids were transfected together with plasmids expressing GR and wild-type or mutant HspBP1 or BAG-1M at the indicated amounts. 24 h after transfection, cells were treated with hormones at the indicated concentrations. In the left panel, the vector control (i.e. “empty” expression vector) was set to 100% (dashed line). In the right panel, the vector control at the highest concentration of hormone was arbitrarily set to 100%. Error bars indicate the standard error of the mean (+SEM) of four independent experiments performed in triplicates (A). Symbols indicate significant differences to vector control for BAG-1M (+), BAG1M_mut ($), HspBP1 (*), and HspBP1_mut (#), with p<0.05, 0.01, 0.001 for one, two or three symbols, respectively. Representative Western controls are shown in B and C for the indicated conditions.

GR activity was impaired by higher levels of both BAG-1M and HspBP1, while at lower levels only BAG-1M exerted a significant stimulatory effect (Fig. 3A, left panel). The mutants of HspBP1 and BAG-1M retained slight inhibitory effects on GR, possibly due to residual Hsp70 binding (Fig. 1). The inhibitory effect of HspBP1 and BAG-1M also showed hormone concentration dependence (Fig. 3A, right panel). Wild-type and mutant NEF proteins were expressed at comparable levels and did not affect GR protein levels (Fig. 3B and C).

**Figure 4:**
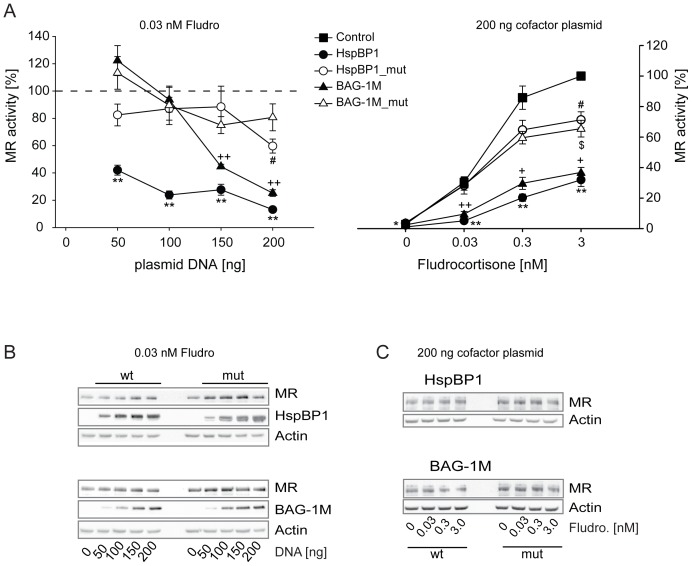
Effects of HspBP1 and BAG-1M on MR transactivation. The impact of HspBP1 and BAG-1M on GR function was assessed by reporter gene experiments in Cos-7 cells. Control and reporter plasmids were transfected together with plasmids expressing MR and wild-type or mutant HspBP1 or BAG-1M at the indicated amounts. 24 h after transfection, cells were treated with hormones at the indicated concentrations. In the left panel, the vector control (i.e. “empty” expression vector) was set to 100% (dashed line). In the right panel, vector control at the highest concentration of hormone was arbitrarily set to 100%. Error bars indicate the standard error of the mean (+SEM) of four independent experiments performed in triplicates (A). Symbols indicate significant differences to vector control for BAG-1M (+), BAG1M_mut ($), HspBP1 (*), and HspBP1_mut (#), with p<0.05, 0.01, 0.001 for one, two or three symbols, respectively. Representative Western blots are shown in B and C for the indicated conditions.

Also MR activity was reduced by higher levels of both BAG-1M and HspBP1 although again BAG-1M at the lowest level was slightly stimulatory (Fig. 4A, left panel). The MR showed a higher sensitivity to HspBP1 than to BAG-1M. Mutants of HspBP1 and BAG-1M also had slight effects on MR activity, very likely due to the residual binding to Hsp70. We observed the strongest inhibitory effect of both wild type NEFs on MR at 0.03 and 0.3 nM fludrocortisone (Fig. 4A, right panel). MR protein levels were unaffected by the two NEFs (Fig. 4B and C).

**Figure 5:**
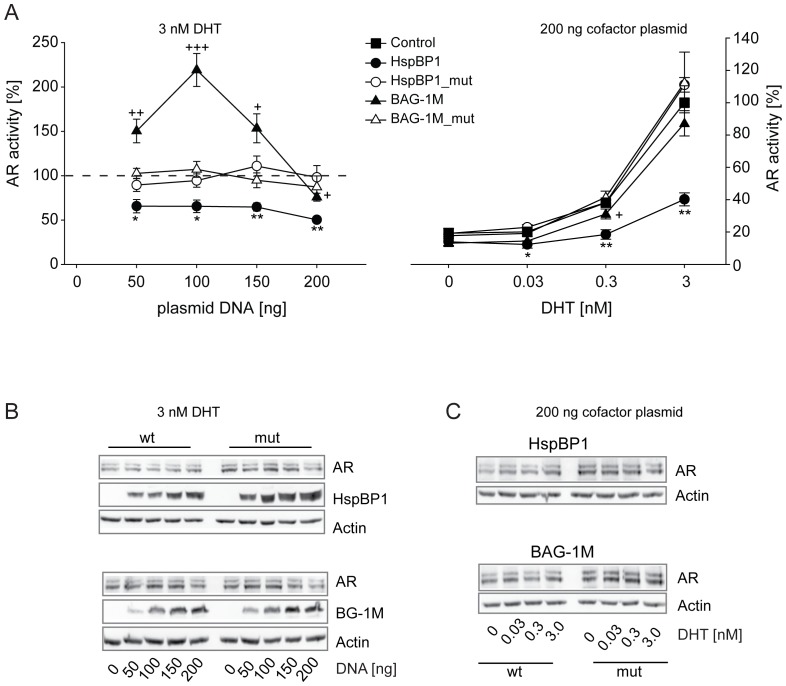
Effects of HspBP1 and BAG-1M on AR transactivation. The impact of HspBP1 and BAG-1M on GR function was assessed by reporter gene experiments in Cos-7 cells. Control and reporter plasmids were transfected together with plasmids expressing AR and wild-type or mutant HspBP1 or BAG-1M at the indicated amounts. 24 h after transfection, cells were treated with hormones at the indicated concentrations. In the left panel, the vector control (i.e.“empty” expression vector) was set to 100% (dashed line). In the right panel, vector control at the highest concentration of hormone was arbitrarily set to 100%. Error bars indicate the standard error of the mean (+SEM) of four independent experiments performed in triplicates (A). Symbols indicate significant differences to vector control for BAG-1M (+), BAG1M_mut ($), HspBP1 (*), and HspBP1_mut (#), with p<0.05, 0.01, 0.001 for one, two or three symbols, respectively. Representative Western blots are shown in B and C for the indicated conditions.

The AR exhibited a markedly different response to BAG-1M than to HspBP1. While HspBP1 diminished AR activation already at low levels, BAG-1M exerted a clear stimulatory effect, which shifted to mild reduction of AR activity at higher BAG-1M expression levels (Fig. 5A, left panel). Hsp70 binding-defective mutants of HspBP1 or BAG-1M had no effect on AR activity. Titration of the AR hormone dihydrotestosterone (DHT) revealed the strongest inhibition of AR by HspBP1 at 3 nM DHT (Fig. 5A, right panel). In contrast, there was only weak AR inhibition by BAG-1M at 3 nM DHT. The divergent impact of the two Hsp70 NEFs on AR could not be explained by effects on AR expression which remained unaffected (Fig. 5B and C).

Since the highest concentrations of hormone used (see Figures 3 to 5) are considered to approach saturating conditions, the results from these reporter gene assays suggest that BAG-1M and HspBP1 have effects other than on hormone binding affinity. In a second set of reporter gene assays, we used higher concentrations of hormones for GR and AR. The inhibitory actions of BAG-1M and HspBP1 on GR at hormone saturation were confirmed (Figure SI-1A). In the case of AR, again only HspBP1 exhibited an inhibitory effect on AR, which was also observed at hormone saturation (Figure SI-1B).

## Discussion

Hsp90- and Hsp70-mediated protein folding events are key determinants of steroid receptor function. While the impact of various Hsp90 cofactors on steroid receptors has been widely investigated [3], [6], cofactors of Hsp70 such as HspBP1 and BAG-1M have received less attention. In this study, we describe for the first time that HspBP1 alters the transcriptional activity of steroid receptors; we also observed mechanistic and functional differences between HspBP1 and BAG-1M in their capacity to shape heterocomplex composition and influence GR, MR, and AR action.

HspBP1 inhibited all three analyzed steroid receptors, i.e., GR, MR, and AR, at a wide range of HspBP1 protein levels. In contrast, BAG-1M exhibited a switch from stimulatory to inhibitory actions on steroid receptors depending on the BAG-1M protein levels. This may be explained by the following molecular mechanisms: at low levels of BAG-1M, the stimulation of the Hsp70 ATPase results in facilitating receptor folding, while at high levels of BAG-1M, increased substrate release overruns subsequent substrate processing, producing an overall inhibitory effect. Another possible mechanism relevant at higher levels of BAG-1M might be based on the inhibition of Hop binding to Hsp70 [3], [32]. This would prevent the folding cycle from proceeding to later stages, i.e., client unloading from Hsp70 and transfer to Hsp90, eventually resulting in steroid receptor inhibition [3], [33]. In support of this hypothesis, our interaction analysis in Fig. 1 revealed only minute amounts of Hop co-precipitating with BAG-1M, and no interaction of BAG-1M with Hip, which is in line with previous reports [13], [16], [34]. Of note, BAG-1M also showed a switch from inhibition to stimulation of Hsp70-mediated luciferase refolding *in vitro* under certain conditions [35].

While stimulatory actions of BAG-1M on GR-dependent transcriptional activity are reported here for the first time, enhancement of the AR and estrogen receptor (ER) activity by BAG-1L are already known [22], [36], [37]. BAG-1M was found to stimulate AR only after forced nuclear expression [38]; here, we observed that BAG-1M mediated AR stimulation occurred in the absence of a nuclear localization sequence in BAG-1M. This seeming contradiction might be explained by varying subcellular distributions of BAG which depend on the investigated cell type [39].

Due to the absence of Hsp40 in HspBP1 complexes, we propose that HspBP1 interferes at an earlier stage of the folding cycle than BAG-1M. Hsp40 is considered to be the first chaperone to bind the steroid receptors and to cooperate later with Hsp70 in protein folding [3], [11]. Thus, prevention of the coordination between Hsp70 and Hsp40 in receptor folding by HspBP1 could contribute to HspBP1’s inhibitory effect on receptor activity. Further support for HspBP1’s impairment of receptor maturation at an early stage comes from the observation that HspBP1 complexes contain very low levels of Hsp90, the core protein of the final receptor folding platform.

Only some aspects of HspBP1 function might be evolutionary conserved, while others are not. In yeast, ATP hydrolysis of the Hsp70 homologue Ssa1p is facilitated by the HspBP1 homologue Fes1p [16], and moreover, substrate binding to the Hsp40 homologue Ydj1p is reduced in the presence of Fes1p [16]. In mammalian cells, HspBP1 was reported to inhibit Hsp40-promoted ATP hydrolysis of Hsp70 [15]. Similarly, HspBP1 was reported to inhibit the ubiquitin E3-ligase CHIP in mammalian cells [40], while the yeast homologue Fes1 was recently described as essential for ubiquitin-dependent degradation of Hsp70 clients [41]. These differences might be due to variations in experimental design or due to the fact that yeast does not replicate all aspects of HspBP1’s function in mammalian cells. The different cellular protein endowment could produce a seeming divergence in function of some proteins between yeast and mammals, as suggested for the effects of the Hsp90 cochaperones FKBP51 and FKBP52 on GR activity [29], [42].

From our experiments in mammalian cells and previous experiments in yeast, the possibility cannot be excluded that the reduction in Hsp40 co-precipitating with HspBP1 is due to reduction in co-precipitating receptor rather than reduced binding to Hsp70. However, experimental evidence has been provided that argues in favor for a direct competition of Hsp40 and HspBP1 for binding to Hsp70 [43]. As a possible consequence, substrate transfer from Hsp40 to Hsp70 could be impaired forming the basis for the here observed reduced client protein binding to Hsp70 in the presence of HspBP1.

A more recent study alerted to the influence of the cellular (co)chaperone composition when analyzing the function of a single protein [7]; it also found that the Hsp40 homologues Hdj2/DNAJA1, Hdj3/DNAJA2, and DNAJA4 exhibit higher binding to MR than to GR. This might relate to our observation that the Hsp40-competing HspBP1 inhibited MR already at lower levels, while higher HspBP1 levels were needed to produce the same inhibitory effect on GR.

Change in hormone binding affinity has long been viewed as the prototypic action of chaperones on steroid receptors. However, evidence has also been provided for additional mechanisms of the Hsp90-based chaperone machinery as well as of BAG-1M that determine heterocomplex composition and assembly on chromatin [44]–[46]. Our observation of inhibitory effects of HspBP1 and BAG-1M on steroid receptors also at saturating hormone concentrations support the notion that the regulation reaches beyond hormone potency, i.e. also affects hormone efficacy possibly involving transcriptional events. For BAG-1M, we also found an inhibitory effect at hormone saturation on the progesterone receptor [23].

Reports on the physiological role of HspBP1 are scarce, and regulation of steroid receptor physiology has not been addressed until now. Major changes in the expression level of HspBP1 have been found in certain cancer cell lines and indicate that HspBP1 might play a role in tumor biology [47], [48]. Since physiological functions of BAG-1 have been linked both to cancer and to steroid receptor activity, such as dexamethasone induced apoptosis [49], hormone-dependent tumors, or stress-related phenotypes [50], [51], our study suggests that more research is needed to explore whether HspBP1 may play a particular role in steroid-dependent tumors and, similar to BAG-1, in stress hormone physiology.

## Supporting Information

Figure S1 **The impact of HspBP1 and BAG-1M on GR and AR function at higher concentrations of hormone was assessed by reporter gene experiments in Cos-7 cells (analogous to** **Figures 3** **and** **5** **).** Control and reporter plasmids were transfected together with plasmids expressing GR (A) or AR (B) and wild-type or mutant HspBP1 or BAG-1M. 24 h after transfection, cells were treated with hormones at the indicated concentrations. The Firefly luciferase activity of the control was arbitrarily set to 100% for 100 nM cortisol (A) or 3 nM DHT (B) (analogous to Figures 3 and 5). Error bars indicate the standard error of the mean (+SEM) of four independent experiments performed in triplicates (A). Symbols indicate significant differences to vector control for BAG-1M (+) and HspBP1 (*), with p<0.05, 0.01, 0.001 for one, two or three symbols, respectively.
(TIF)
