# Supplementary figures and images for: Hsp70 Cochaperones HspBP1 and BAG-1M Differentially Regulate Steroid Hormone Receptor Function
Source: PLoS One. 2014 Jan 14;9(1):e85415. doi: 10.1371/journal.pone.0085415 (PMC3891853; doi:10.1371/journal.pone.0085415)

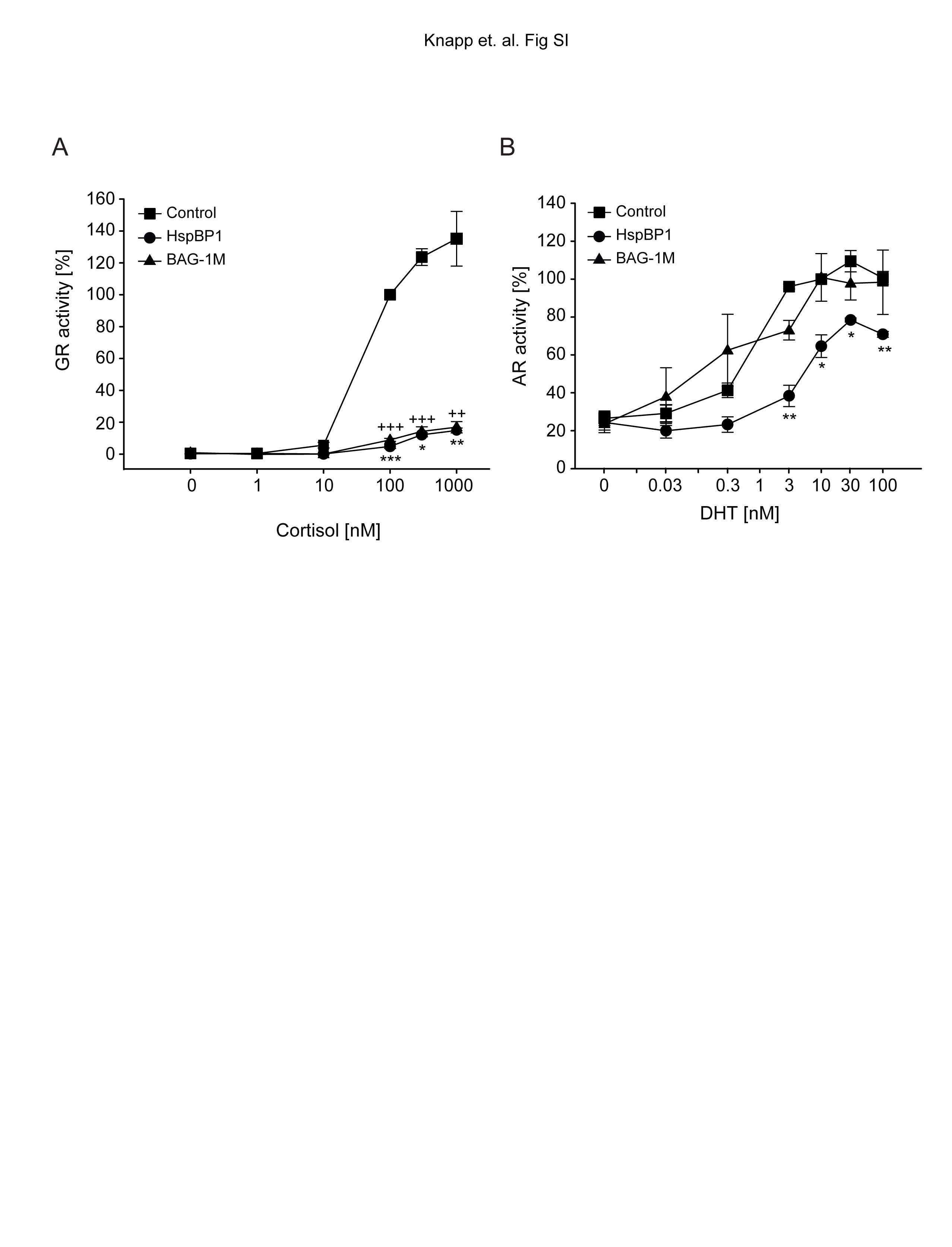

Supplement: Figure S1 — The impact of HspBP1 and BAG-1M on GR and AR function at higher concentrations of hormone was assessed by reporter gene experiments in Cos-7 cells (analogous to Figures 3 and 5 ). Control and reporter plasmids were transfected together with plasmids expressing GR (A) or AR (B) and wild-type or mutant HspBP1 or BAG-1M. 24 h after transfection, cells were treated with hormones at the indicated concentrations. The Firefly luciferase activity of the control was arbitrarily set to 100% for 100 nM cortisol (A) or 3 nM DHT (B) (analogous to Figures 3 and 5). Error bars indicate the standard error of the mean (+SEM) of four independent experiments performed in triplicates (A). Symbols indicate significant differences to vector control for BAG-1M (+) and HspBP1 (*), with p<0.05, 0.01, 0.001 for one, two or three symbols, respectively. (TIF) [file pone.0085415.s001.tif]
